# Docosahexaenoic Acid (DHA) Supplementation in a Triglyceride Form Prevents from Polyglutamine-Induced Dysfunctions in *Caenorhabditis elegans*

**DOI:** 10.3390/ijms252312594

**Published:** 2024-11-23

**Authors:** Ignasi Mora, Alex Teixidó, Rafael P. Vázquez-Manrique, Francesc Puiggròs, Lluís Arola

**Affiliations:** 1 Brudy Technology S.L., 08006 Barcelona, Spain; cultivos1@brudylab.com; 2 Universitat Rovira i Virgili, 43003 Tarragona, Spain; 3 Eurecat, Centre Tecnològic de Catalunya, Nutrition and Health Unit, 43204 Reus, Spain; alex.teixido@estudiants.urv.cat; 4 Laboratory of Molecular, Cellular and Genomic Biomedicine, Instituto de Investigación Sanitaria La Fe, 46026 Valencia, Spain; rafael_vazquez@iislafe.es; 5 Joint Unit for Rare Diseases, Insituto de Investigación Sanitaria La Fe-Centro de Investigación Príncipe Felipe, 46012 Valencia, Spain; 6 Centro de Investigación Biomédica en Red de Enfermedades Raras (CIBERER), 28029 Madrid, Spain; 7 Eurecat, Centre Tecnològic de Catalunya, Biotechnology Area, 43204 Tarragona, Spain; 8 Nutrigenomics Research Group, Departament de Bioquímica i Biotecnologia, Universitat Rovira i Virgili, 43007 Tarragona, Spain; lluis.arola@urv.cat

**Keywords:** polyQ, DHA, omega-3, Huntington’s, *C. elegans*, serotonin

## Abstract

A common hallmark of neurodegenerative diseases is the accumulation of polypeptide aggregates in neurons. Despite the primary cause of these diseases being inherently genetic, their development can be delayed with proper preventive treatments. Long-chain polyunsaturated fatty acids (ω-3 LCPUFA) are promising bioactive nutrients that are beneficial for brain health. In this study, the impact of an oil rich in a structured form of docosahexaenoic acid (DHA) triglyceride (TG) was assessed in a *Caenorhabditis elegans* model expressing long poly-glutamine (polyQ) chains, which mimics the symptomatology of polyQ-related neurodegenerative diseases such as Huntington’s disease (HD), among others. The lifespan, the motility, the number of polyQ aggregates, the oxidative stress resistance, and the cognitive performance associated with sensitive stimuli was measured in mutant nematodes with polyQ aggregates. Overall, DHA-TG at 0.5 µM improved the lifespan, the motility, the oxidative stress resistance, and the cognitive performance of the nematodes, emphasizing the protection against serotonergic synapse dysfunction. Furthermore, the treatment reduced the polyQ aggregates in the nematodes. The data described herein shed light on the connection between DHA and the cognitive performance in neurodegenerative diseases and demonstrated the potential of DHA-TG as nutritional co-adjuvant to prevent the development of polyQ-associated dysfunctions.

## 1. Introduction

A common pathological hallmark of the most prevalent neurodegenerative diseases like Parkinson’s disease (PD), Alzheimer’s disease (AD), Huntington’s disease (HD), and spinocerebellar ataxias (SCA), among many others, is the accumulation of lipo-protein and protein aggregates in the neurons. These aggregates are caused by genetic mutations and impair the right functions of neurons through disrupting many key cell processes by producing cytotoxic effects such as neuroinflammation (inflammation of the nervous system) and oxidative stress, which further aggravate protein aggregation and neurodegeneration [1,2].

Huntington disease is a dominant hereditary neurodegenerative disease caused by a CAG trinucleotide repeat which encodes tracts of polyglutamines (polyQs) in the gene encoding the Huntingtin (Htt) protein. Its mutation leads to the generation of mutant Htt (mHtt) with an abnormally long polyQ-chain at the amino terminal of the protein. These amino-terminal fragments play a key role in the aggregation of mHtt, which eventually develops in inclusion bodies [3]. The physiological role of Htt is not completely understood, but its function seems essential for life in mammals [4]. As mentioned, the disease caused by polyQ repeats also include six of the SCAs, dentatorubral–pallido-luysian atrophy (DRPLA), and spino-bulbar muscular atrophy (SBMA) [5]. Those who suffer from these diseases manifest deficits in motor function, progressive cognitive decline, and severe psychiatric disturbances such as depression and mood swings [6]. Despite its well-defined genetic origin, the molecular and cellular mechanisms underlying these polyQ-related diseases are unclear and complex [7] and most importantly, they lack a cure or a treatment that delays the progression of the disease.

Although the primary cause of neurodegenerative diseases is inherently genetic, some strategies can be developed to tackle the main risk factors leading to the early onset of these pathologies, such as dysregulated protein homeostasis in the cells [8], increased neuroinflammation or high levels of oxidative stress [7]. In this sense, preventive nutritional treatments and bioactive ingredients are gaining attention for promoting brain health in aged individuals since diet has been demonstrated to modulate the risk factors leading to cognitive dysfunctions [9].

Among bioactive compounds for brain health, ω-3 long-chain polyunsaturated fatty acids (ω-3 LCPUFA), mainly docosahexaenoic acid (DHA, C22:6n-3) and eicosapentaenoic acid (EPA, C20:5n-3), are involved in multiple biochemical functions of the nervous system, including the synthesis of endogenous metabolites, cell membrane fluidity, intracellular signaling, and the regulation of transcription factors [10]. Moreover, DHA could enhance the activity of antioxidant enzymes, improving the capacity of the cell to detoxify and counteract oxidative stress [11], which might minimize age-related inflammation consequences [12]. In cognitive function, the increased intake of DHA and EPA has been associated with better cognitive performance, slower rates of cognitive decline, and an overall lower risk of developing dementia [13]. In addition, DHA and EPA might be effective delaying the development of AD and PD in patients at the early stage of the diseases [14]. In polyQ-related diseases, the effect of ω-3 LCPUFA on symptom control and disease progression has not been fully examined because untargeted research has been carried out; however, indirect evidence suggests that DHA and EPA might help to alleviate polyQ-related symptoms, since they provide proven reduction in cachexia and weight loss, together with the prevention of cognitive decline over time, which are common traits of the advanced stages of polyQ-related diseases [15].

The nematode *Caenorhabditis elegans* (*C. elegans*) is a genetically tractable experimental model widely used to study aging and neurodegenerative diseases, including the ones with polyQ aggregates. Worms expressing polyQs in different tissues helped to uncover many genes modulating the rate of aggregation and/or the toxicity induced by these molecules [16]. *C. elegans* use and synthesize LCPUFA, which are thought to be vital for some functions of nematodes, including correct neurotransmission [17] or pharyngeal pumping [18]. In previous research, we proved that the ω-3 LCPUFA, DHA, was able to improve the motility and antioxidant response of nematodes [19], but little is known about its effect on nematodes suffering from polypeptide aggregates. In 2022, SenGupta et al. reported that krill oil supplementation protected dopaminergic neurons in a *C. elegans* Parkinsonism model [20], and recently, Wang et al. reported that EPA and DHA supplementation attenuated the amyloid-beta-induced toxicity in an Alzheimer’s model of *C. elegans* through the activation of the peroxisome proliferator-activated receptor (PPAR)-ɣ [21].

In this study, we used two *C. elegans* models expressing 40Q-chains fused in frame to the yellow fluorescence protein (40Q::YFP), which formed protein aggregation and fluorescent polyQ aggregates. Both of them mimicked HD and SCA traits, among other polyQ-associated diseases, since polyQ sequences longer than 36 residues are correlated with the onset of these diseases [22].

In food matrixes, DHA is present in natural triacylglycerol (TG) forms, with small amounts of other acylglycerols and free fatty acids that are also produced in lipid digestion [23]. However, ω-3 concentrates on the market are usually produced as DHA ethyl-esters (DHA-EE), which are not present in natural sources. The main reason is that from an industrial point of view, the EE structure allows to concentrate easily the DHA of the oils [24]. Nevertheless, most evidence has reported that DHA-EE oils have a reduced bioavailability and tissue membrane incorporation in comparison to DHA-TG oils [25,26]. This finding has motivated the launch of new enzymatically re-esterified TG as alternatives to previously existing products, which have been demonstrated to restore bioavailability and even improve the bioactivity of DHA oils [27]. Our research was carried out using a structured form of DHA triglyceride (DHA-TG), in which the fatty acids were in targeted positions.

In brief, the main purpose of this study was to shed light on the potential of DHA as a nutritional co-adjuvant to improve polyQ-related neurodegenerative diseases from a preventive point of view. Through using a *C. elegans* model expressing 40Q aggregates, we elucidated the impact of an oil rich in DHA-TG on the lifespan, the polyQ accumulation, the motility, the neurotransmission, and the gene expression levels of nematodes.

## 2. Results

### 2.1. Determining DHA Concentration in WT Nematodes

The first step of our research was to set an optimal concentration to treat the nematodes. According to previous studies, concentrations of DHA-TG close to 50 µM provided beneficial effects and were not toxic for *C. elegans* [19]. However, as we were seeking the most bioactive concentration, we tried to lower the doses. With this purpose, the lifespan and the locomotive function after treatment were assessed in the wild-type (WT) nematodes, with a wider range of DHA concentrations. DHA-TG nanoemulsions were used for the treatments since TGs are very insoluble in hydrophilic matrixes such as the nematode growth media (NGM). In previous studies, the method with nanoemulsions was reported as successful for TG treatments [28].

**Figure 1:**
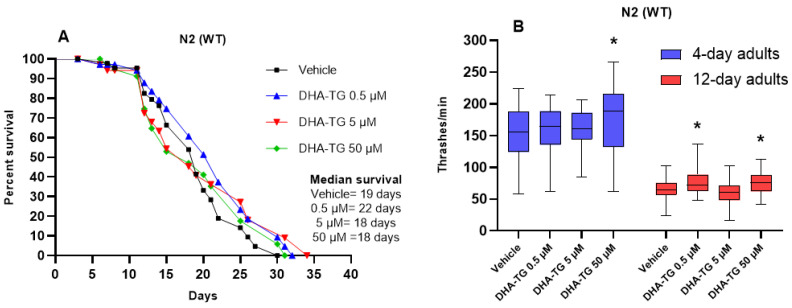
Healthspan effect of DHA-TG in N2 wild-type (WT) nematodes. (**A**) Effect on the lifespan at 20 °C. (**B**) Effect in motility measuring thrashes per minute at the fertile stage (4-day adult) and the aged stage (12-day adult) after treatments; data are represented as boxes showing the median of thrashing, Q1 and Q3 quartiles, and whiskers expressing minimum to maximum values (n > 30 for each condition). The statistical differences compared to the vehicle were considered significant at *p* < 0.05 (*) after a two-way ANOVA followed by Dunnett’s post-test.

Despite not finding any significant effect with the concentrations of 0.5 and 50 µM, the WT nematodes tended to show a prolonged lifespan (Figure 1A). Furthermore, both concentrations improved the motility of aged *C. elegans* (12-day old) (Figure 1B), and curiously, the intermediate concentration, 5 µM, did not produce any motility or lifespan change compared to the control group (vehicle). Consequently, we decided to move forward in our research with 0.5 and 50 µM concentrations of DHA-TG. Studies assessing the effect of polyphenols [29] and PUFA [30] in *C. elegans* have also described a lack of effect with intermediate concentrations since the beneficial effects of bioactive nutrients are sometimes mediated by hormetic adaptations (low-dose stimulation).

### 2.2. Lifespan and Motility Promoting Effect of DHA-TG in AM101 (rmls110) Nematodes

**Figure 2:**
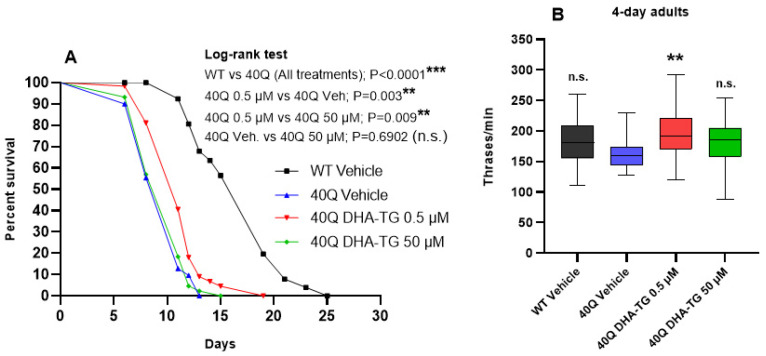
Healthspan effect of DHA-TG in nematode strain AM101 (*rmIs110*[F25B3.3p::Q40::YFP]). (**A**) Effect on the lifespan at 20 °C. Statistical differences in the lifespan were considered significant at *p* < 0.01 (**) and *p* < 0.001 (***) after a Long-rank test of the survival curves vs. the 40Q vehicle. *p*-value is expressed beside the curves (n.s. = not significant). (**B**) Effect in motility measuring thrashes per minute of nematodes at 4-day adult stage after treatments; the data are represented as boxes showing the median of thrashing, Q1 and Q3 quartiles, and whiskers expressing the minimum to maximum values (n > 30 for each condition). Statistical differences compared to the 40Q vehicle were considered significant at *p* < 0.01 (**) after one-way ANOVA followed by Tukey’s test (n.s. = not significant).

As made in WT nematodes, the impact on the lifespan and in the motility was measured in the AM101 (*rmIs110*[F25B3.3p::Q40::YFP]) mutant strain, which expressed 40Q florescent aggregates in the neurons (Figure S1, Supplementary material). This strain shows an impaired lifespan, a deficient motility, and a disrupted neuronal function due to 40Q aggregates in the neurons [31]. The treatment with DHA at 0.5 µM significantly prolonged the lifespan of *rmls110* mutants as illustrated in Figure 2A; however, the lifespan of the mutant (median survival of 11 days) was far from the WT lifespan (median survival of 19 days). Since the median survival of the 40Q nematodes was 11 days, we decided to change the experimental design, and the measurements were just made with nematodes on their 4th day of adulthood.

Agreeing with the effects observed in the lifespan, the DHA-TG concentration of 0.5 µM significantly improved the motility of the *rmls110* mutant nematodes compared to the 40Q control (40Q vehicle). Conversely, the treatment at a concentration of 50 µM did not have a significant effect in the motility (*p*-value = 0.07) (Figure 2B). When using polyQ-models, the increase in thrashes was corelated with a better psychomotor function and potentially with an improvement of the dysfunctions caused by polyQ aggregates [16]. According to the references, ω-3 PUFA treatments already provided motility improvement in a rodent model of HD [32].

### 2.3. PolyQ Reduction After DHA-TG Treatment in AM141 (rmIs133) Nematodes

**Figure 3:**
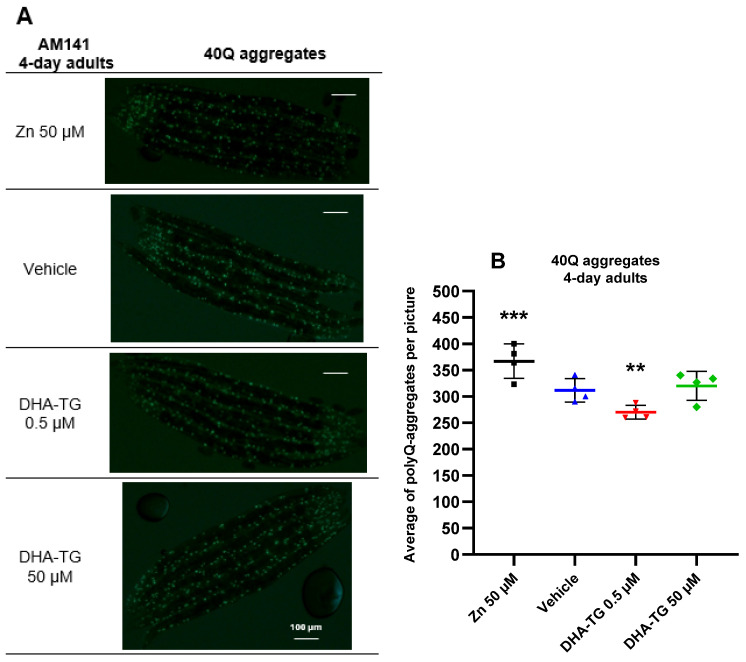
(**A**) Pictures of AM141 nematodes (*rmIs133* [unc-54p::Q40::YFP]) on the 4th day of adulthood after treatments. Worms were mounted onto 2% agar pads and anesthetized with a drop of 0.5 M sodium azide. Images were taken using a DM2500 (Leica, Wetzlar, Germany) vertical fluorescence microscope. (**B**) Number of 40Q aggregates in the AM141 *rmIs133*[unc-54p::Q40::YFP]) strain nematodes at 4 days of adulthood after treatments. Data shows a line expressing the average of 40Q aggregates per picture ±SD counted in 4 different pictures. Each point (4 points per treatment) represents an average of 40Q aggregates per picture. Each picture had 5 worms for a total n = 20. Fluorescent 40Q aggregates were counted with ImageJ. Differences were considered significant at *p* < 0.01 (**) and *p* < 0.001 (***) after one-way ANOVA followed by Tukey’s test.

For assessing the polyQ aggregates’ reduction a strain expressing 40Q::YFP in muscular cells was used: the AM141 strain (*rmIs133*[unc-54p::Q40::YFP]). Since polyQs are expressed in muscular cells they can be counted clearly, and the number of inclusion bodies that are an indirect measurement of the aggregation process can be more precisely determined (Figure S1). An extra treatment with ZnCl_2_ at 50 µM was included for *rmIs133* mutant nematodes as a positive control, since Zn^2+^ exposure causes toxic effects, increasing polyQ aggregates [33], altering locomotion, and disrupting behavior in polyQ nematodes [34]. Since the number of 40Q aggregates grows with age, positive control with zinc significantly increased the polyQ aggregates in 4-day old nematodes (Figure 3), but there was not a significant increase at the young-adult stage (Figure S2).

The treatment with DHA-TG at 50 µM significantly reduced the 40Q aggregates at the young-adult stage (Figure S2), but did not reduce the polyQs in 4-day old nematodes (Figure 3). On the contrary, the DHA-TG at 0.5 µM efficiently reduced the 40Q aggregates in 4-day old nematodes (Figure 3). Since our intervention was mainly focused on the advanced stages of polyQ pathology (4-day adults), the dose of 0.5 µM was determined as the most effective concentration, fighting against the synthesis of 40Q aggregates. Furthermore, the effect of DHA-TG at 0.5 µM at advanced stages of the diseases correlates with the enhanced lifespan observed in Figure 2A. Consequently, in the following experiments, we only used the concentration of 0.5 µM of DHA, and the 40Q aggregates results correlated with the ones of motility (Figure 2B).

**Figure 4:**
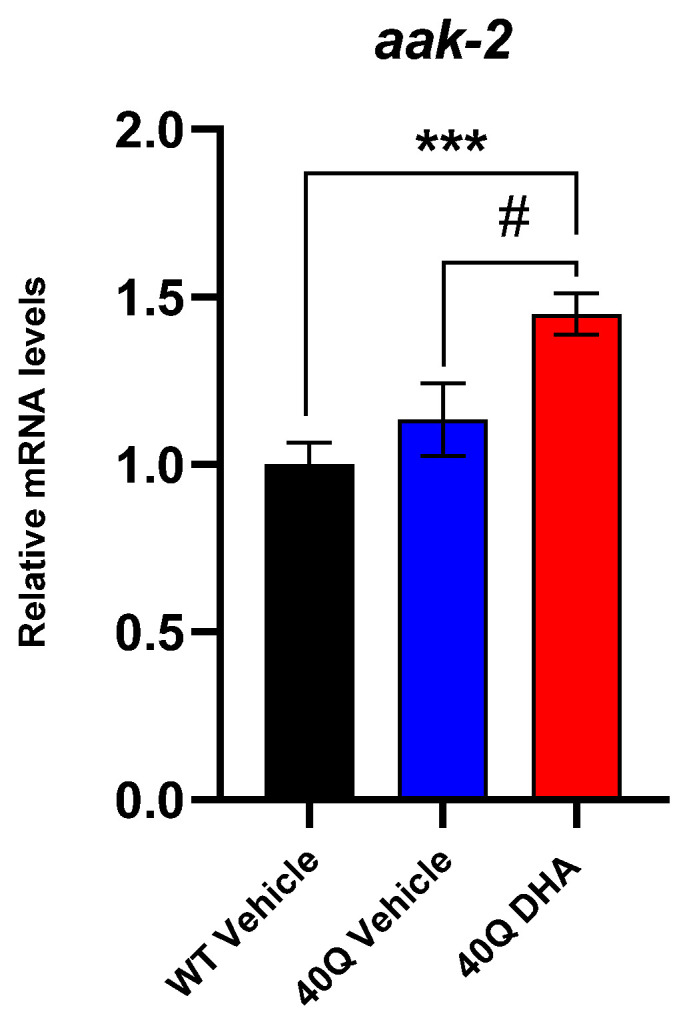
Expression levels of *aak-2* in AM101 mutant nematode at 4 days of adulthood after DHA-TG 0.5µM treatment expressed vs. WT. statistical differences compared to WT vehicle are expressed with (*), and statistical differences compared to 40Q vehicle are expressed as (#). Differences were considered significant at *p* < 0.001 (***) and *p* < 0.05 (#) after one-way ANOVA followed by Tukey’s test.

In addition to recording the polyQ aggregates, the expression level of some key genes related to polyQ aggregate reduction was also assessed. Unfortunately, the DHA-TG failed to promote the transcription of the genes related with polyQ reduction, such as sir-2.1/SIRT 1 (Figure S3) or daf-16/FOXO (Figure S4) [35], but the gene expression of *aak-2*, which encodes for a subunit of adenosine–monophosphate-activated protein kinase (AMPK), AMPKα2, was remarkably increased (Figure 4). AMPKα2 is one of the two proteins forming the catalytic core of AMPK, a kinase that controls the energy homeostasis in the cell and phosphorylates a wide range of proteins in response to metabolic changes, mostly ATP fluctuations. After activation, AMPK can promote the initiation of autophagy through the inhibition of the mammalian target of rapamycin (mTOR) and other intermediates, under conditions of low levels of ATP or stress of different kinds [36]. In the brain, AMPK activation might protect against HD by inducing pro-survival pathways that prevent the cytotoxicity induced by Huntingtin with polyQ aggregates, and *aak-2* activation is essential for the protective effects of AMPK against polyQ by mediating aggregate reduction in *C. elegans* and rodent models [37]. This result suggests that DHA-TG treatment might contribute to the reduction in polyQ through activating *aak-2* gene expression (Figure 4) and consequently promoting AMPK activity.

### 2.4. Oxidative Stress Resistance of AM101 (rmls110) Nematodes After DHA-TG Treatment

*C. elegans* polyQ-models are theoretically described as hypersensitive to oxidative stress. They usually show an upregulated expression of antioxidant defense genes due to the increased levels of ROS (reactive oxygen species) caused by polyQ aggregates. ROS aggravate polyQ-related diseases, but they are not the cause of the diseases [38]. Since DHA-TG has provided antioxidant effects [13], we wanted to assess whether the treatment could be able to improve the oxidative stress resistance of the mutant strain AM101 (*rmIs110*[F25B3.3p::Q40::YFP]) with pan-neuronal 40Q aggregates.

**Figure 5:**
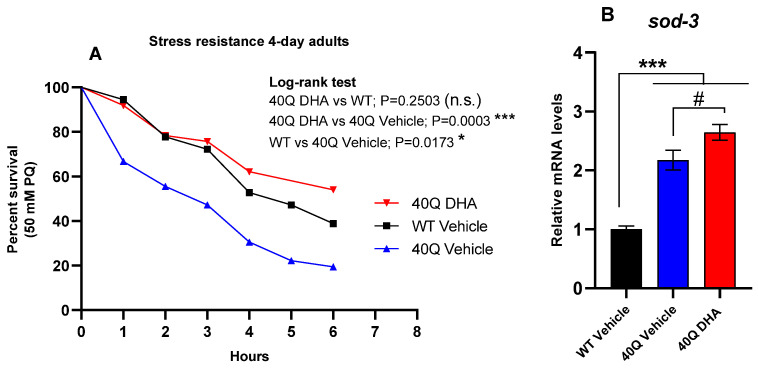
Stress resistance capacity of nematode strain AM101 (*rmIs110*[F25B3.3p::Q40::YFP]) after DHA-TG 0.5µM treatment. (**A**) Survival curves at 20 °C inducing ROS with 50 mM PQ solution (n > 30 for each condition). Statistical differences considered significant at *p* < 0.05 (*) and *p* < 0.001 (***) 8 after Long-rank test of survival curves. *p*-value and tests are detailed beside curves (n.s. = not significant). (**B**) Expression levels of *sod-3* in AM101 mutant nematodes at 4 days of adulthood expressed vs. WT. statistical differences compared to WT vehicle are expressed with (*), and statistical differences compared to 40Q vehicle are expressed as (#). Differences were considered significant at *p* < 0.05 (#) and *p* < 0.001 (***) after one-way ANOVA followed by Tukey’s test.

The treatment with DHA successfully improved the resistance to oxidative damage, as is shown in Figure 5A. In fact, the resistance to the paraquat (PQ) of the mutant strain with DHA was close to the WT capacity. Moreover, the DHA treatment increased the sod3 gene expression (Figure 5B) compared to the 40Q strain (40Q vehicle), thus supporting mitochondrial SOD activity. On the contrary, the expression of daf-16 and skn-1 genes, which encode important transcription factors regulating antioxidant defense, was not increased (Figure S4). Supporting our results, a study with a *C. elegans* 40Q-model also found that the treatment with organoselenium compounds improved ROS counteraction through the overexpression of *sod-3* [39].

### 2.5. Neurobehavioral Function After DHA-TG Treatment of AM101 (rmls110) Nematodes

#### 2.5.1. Dopaminergic Response

**Figure 6:**
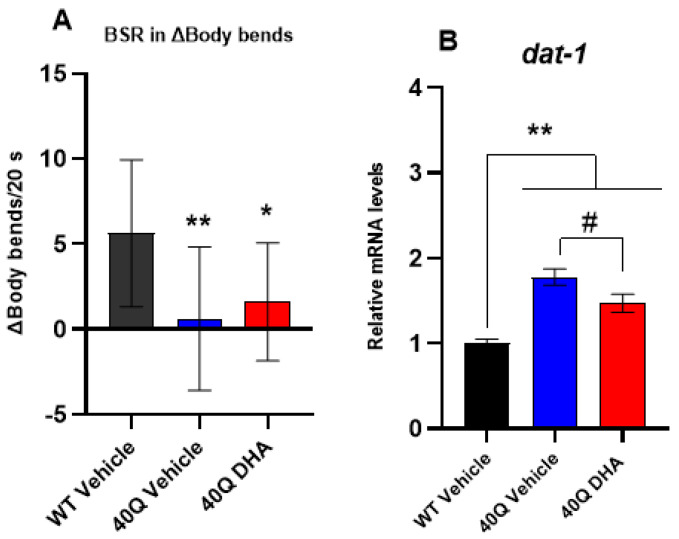
Dopaminergic response of nematode strain AM101 (*rmIs110*[F25B3.3p::Q40::YFP]) at 4 days of adulthood after DHA-TG 0.5 µM treatment. (**A**) BSR represented as Δbody bends per 20 s. The data are represented as boxes showing the median of Δbody bends, Q1 and Q3 quartiles, and whiskers expressing the minimum to maximum values (n > 12 for each condition). Statistical differences compared to WT vehicle were considered significant at *p* < 0.05 (*) and *p* < 0.01 (**) after one-way ANOVA followed by Tukey’s test. (**B**) Expression levels of *dat-1* in AM101 mutant nematodes expressed vs. WT. statistical differences compared to WT vehicle are expressed with (*), and statistical differences compared to 40Q vehicle are expressed as (#). Differences were considered significant at *p* < 0.05 (#) and *p* < 0.01 (**) after one-way ANOVA followed by Tukey’s test.

When the dopaminergic synapse is normal, the locomotion of worms slows down if food is present. This behavior is known as basal slowing response (BSR). For BSR measurement, the worm’s body bends are counted for 20 s and the locomotion is quantified with and without food, as expressed in Figure S5. To emphasize the comparison of BSR between the groups, it can be expressed as Δbody bends for 20 s. (subtracting body bends with food from body bends without food), as in Figure 6A. Higher Δbody bends for 20 s. means greater BSR and better dopaminergic response [40].

According to our data (Figure 6A), the pan-neuronal 40Q strain has an unpaired dopaminergic connection, which agrees with the symptomatology of HD patients [41]. The treatment with DHA-TG did not recover the dopaminergic function since the Δbody bends did not reach the levels of the WT nematodes and there were no significant differences compared to the 40Q vehicle group. However, with the treatment of DHA-TG, the gene expression of *dat-1*, which encodes the dopamine transporter (DAT), was significantly reduced in comparison to the 40Q control. DAT is a dopamine (DA) transporter that removes DA from the synaptic cleft, thus terminating the signal of the neurotransmitter. When DAT is dysfunctional or inhibited, the dopaminergic neurons are in a permanent excitatory state. Conversely, DAT overexpression is correlated with the destruction of dopaminergic neurons since it favors the accumulation of DA and toxic substances inside the pre-synaptic neurons, which cause cytotoxic effects [42]. DA levels follow a biphasic pattern in HD patients, with increased levels in the early stages causing hyperkinesia (excessive abnormal movements), and decreased concentrations in the later stages of the disease causing hypokinesia (inability to produce movement) [43]. Pre-clinical studies suggested DHA might contribute to recovering the normal function of DA neurons through slowing down the DA metabolism in Parkinsonism-like models [44], and DAT downregulation might be an indicator of slowed DA metabolism [45]. However, as the regulation of DA in HD patients is not completely understood, and little information is known in *C. elegans* models, the downregulation of *dat-1* cannot be determined as beneficial.

#### 2.5.2. Serotonergic Response

As previously mentioned, the healthy worms shortly deprived of food moved significantly slower in food presence than in BSR (as shown in Figure S6); this behavior is known as enhanced slowing response (ESR) [46]. The worms deprived of food became more sensitive to detecting bacteria, and their movements slowed down as an associative learning response to the short starvation period (30 min). Serotonin (5-HT) in sensitive neurons regulates their olfactory preferences, which are suggestive of associative learning [47].

**Figure 7:**
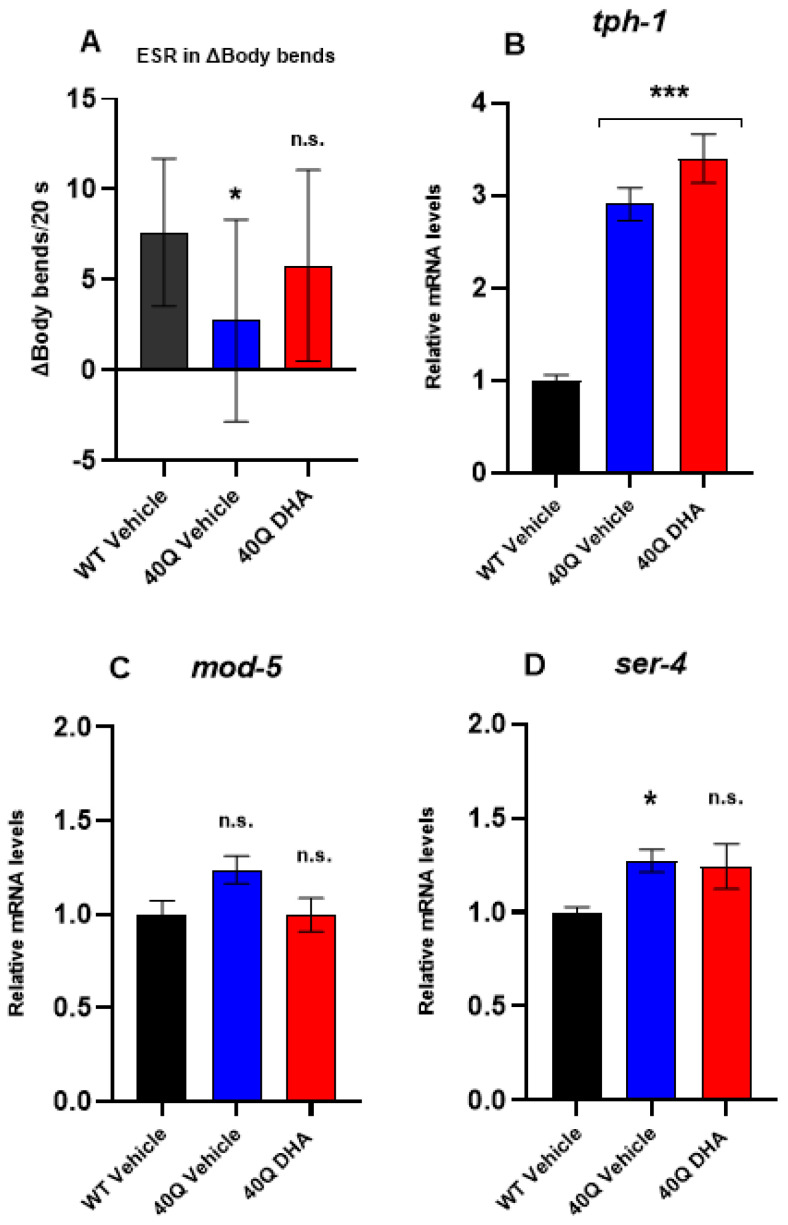
Serotonergic response of nematode strain AM101 (*rmIs110*[F25B3.3p::Q40::YFP]) at 4 days of adulthood after DHA-TG 0.5 µM treatment. (**A**) ESR represented as Δbody bends per 20 s. The data are represented as boxes showing the median of Δbody bends, Q1 and Q3 quartiles, and whiskers expressing the minimum to maximum values (n > 12 for each condition). Statistical differences compared to WT vehicle were considered significant at *p* < 0.05 (*) after one-way ANOVA followed by Tukey’s test (n.s. = not significant). (**B**) Gene expression levels of ser4 in AM101 strain vs. WT. (**C**) Gene expression levels of mod5 in AM101 strain vs. WT. (**D**) Gene expression levels of tph1 in AM101 strain vs. WT. In (**B**–**D**) statistical differences compared to WT vehicle were considered significant at *p* < 0.05 (*) and *p* < 0.001 (***) after one-way ANOVA followed by Tukey’s test (n.s. = not significant).

The comparison of ESR between groups is expressed as Δbody bends for 20 s in Figure 7A, subtracting the body bends with food from the body bends without food (complete data in Figure S5B), as with BSR (Figure 6A). Higher Δbody bends for 20 s means greater ESR and better serotonergic response.

The pan-neuronal 40Q strain showed a defective ESR since the Δbody bends were significantly lower than the WT, as shown Figure 7A. In consequence, this polyQ strain expressed an impaired serotonergic response. Conversely, the 40Q nematodes treated with DHA-TG did not show a significant ESR reduction, which means that the DHA-TG treatment prevented the mutants from the development of an impaired serotonergic response.

Additionally, the expression level of some key genes related to 5-HT metabolism was also assessed. The gene *tph-1* encodes the enzyme tryptophan hydroxylase (TPH) and its ortholog gene in humans encodes TPH1 and 2. Surprisingly, *tph-1* is remarkably overexpressed in 40Q nematodes (Figure 7B) in comparison to WT. It seems reasonable that TPH would be overexpressed in the 40Q model trying to compensate for the dysfunctional TPH activity, a common trait of neurodegenerative disease [48]. In fact, it was already described in an HD rodent model that the overexpression of the gene encoding TPH was not correlated with the enzymatic activity of TPH, which was reduced in comparison to healthy individuals [49]. The DHA-TG treatment slightly increased the expression of *tph-1*, but no significant differences were found (Figure 7B).

Also, it was quantified the expression level of *mod-5* (Figure 7C), which encodes SERT (SLC6A4 in humans), a transporter responsible of 5-HT reuptake. The overexpression of SERT potentially causes 5-HT depletion, which is thought to increase anti-social behavior in HD patients [15]. The pan-neuronal 40Q model showed a tendency (*p*-value = 0.06) to increase the expression of *mod-5*, but DHA-TG treatment recovered the WT levels (Figure 7C). Hence, the restored *mod-5* expression, which controlled the 5-HT reuptake, in combination with an increased *tph-1* expression, which promoted 5-HT synthesis, might have promoted the release of 5-HT in the 40Q strain treated with DHA-TG.

Moreover, the DHA-TG treatment slightly reduced the expression of *ser-4* in the 40Q model (Figure 7D). This *ser-4* gene encoded a G protein-coupled 5-HT receptor (ortholog of human HTR1A (5-hydroxytryptamine receptor 1A)), which seemed to be associated with the locomotive function, and in *C. elegans* it was linked with the fast 5-HT-coupled motor response [50]. An increased expression of 5-HT1A, the receptor encoded by the *ser-4* homologous gene was also described in the rodent model of HD [49]. The main hypothesis to explain *ser-4* increasing was that the serotonergic neurons were trying to compensate for the dysfunctional 5-HT signaling through increasing the expression of the 5-HT receptors.

Overall, these results indicate that DHA-TG might stabilize the 5-HT signaling of 40Q nematodes, even increasing the 5-HT release, thus promoting the efficient neurotransmission of serotonergic sensitive neurons and recovering the normal ESR in the 40Q model.

## 3. Discussion

In this study, we proved that supplementation with an oil rich in DHA-TG can improve the lifespan, the motility, the oxidative stress resistance capacity, and the synaptic function of a *C. elegans* mutant strain with pan-neuronal 40Q aggregates, in addition to reducing the 40Q aggregates of a strain expressing 40Qs in muscular cells. Therefore, the DHA-TG treatment was able to alleviate the dysfunctions caused by the neuronal accumulation of polyQ and to reduce the polyQ aggregates significantly, thus preventing the nematodes to develop the symptoms of polyQ-related diseases.

As we already reported in previous findings with WT nematodes [19], the DHA-TG treatment increased the expression of the *sod-3* gene which codes for SOD-3 enzyme, an ortholog of the human gene encoding Mn-SOD (or SOD-2). The enzyme Mn-SOD is mostly present in the mitochondria of the cells. Mitochondria homeostasis disruption is a common trait in the early stages of HD pathology, because polyQ aggregates attack the mitochondria membranes of neurons and affect their proper function [51]. Hence, the promotion of Mn-SOD activity (SOD-3 in *C. elegans*) might protect the mitochondria from developing dysfunctions, since mitochondria is the main source of ROS in the cells, and SODs counteract oxidative stress. Thus, the improvement of polyQ-associated dysfunctions after SOD-3 promoting treatments in *C. elegans* is already reported in the references [39,52].

Despite the Mn-SOD findings and the huge number of studies assessing antioxidants for the treatment of polyQ toxicity, no antioxidant drug nor natural compound has provided a substantial recovery in human patients suffering from polyQ-related diseases. Antioxidants might be able to temporarily reduce the presence of free radicals, but they are incapable of maintaining a sustained reduction in toxic species while polyQ are still growing with aging. Thus, as Bono-Yagüe et al. postulated, the main treatments for polyQ-related disease must be directed primarily to reduce its formation or to improve the proteostasis and the autophagy in neurons [53]. As an example, Machiela et al. [38] already described that increasing oxidative stress in a polyQ worm model did not worsen the deficits caused by polyQ toxicity and the treatment with antioxidants failed to improve the motility of worms with HD.

Consequently, our study was not just focused on reducing the ROS, but also had a global approach on the dysfunctions caused by polyQ aggregates. For this reason, we also measured genes like *aak-2* which encodes AMPKα, one of the subunits of AMPK. The kinase AMPK seems to be pivotal to counteract polyQ-associated dysfunctions in different experimental models like *C. elegans* [16], rodents [37,54], and in clinical trials [4]. However, since AMPK has many functions in metabolism, the exact impact and benefits that it might have in polyQ models, other than increased autophagy and polyQ reduction, are still unknown [36].

Furthermore, we reported that the nematodes expressing pan-neuronal 40Q have an impaired dopaminergic and serotonergic sensitive response, which is reasonable due to the disrupted neuronal function caused by the aggregates in these worms [55], and we found that DHA-TG treatment successfully prevented 40Q nematodes from developing an impaired serotonergic response. Previous studies showed that DHA recovered the normal synaptic function in PUFA-depleted *C. elegans* mutants [56], and it was already reported that krill oil supplementation protected dopaminergic neurons in a *C. elegans* Parkinsonism model [20]. Nevertheless, for the first time, it has been reported that DHA can prevent serotonergic synaptic dysfunction in a neurodegenerative disease nematode model.

**Figure 8:**
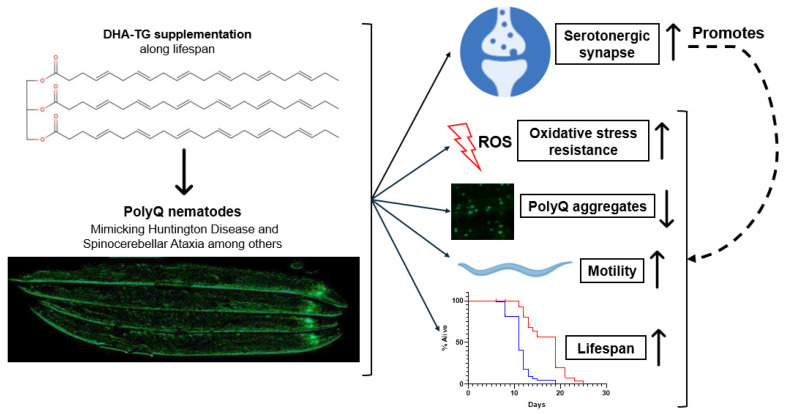
Effects of DHA-TG on nematode’s mutant strains with polyQ aggregates.

The relevance of our findings lie in the importance of 5-HT as a neuroendocrine messenger of environmental conditions for regulating the motility and metabolism of nematodes [50,57]. Berendzen et al. demonstrated that the neuronal mitochondrial dysfunction produced by polyQ can affect 5-HT release, and the lack of proper 5-HT signaling can modulate the mitochondrial function in distal tissues by making them more prone to polyQ toxicity [58]. Furthermore, different studies reported that 5-HT can improve resistance to oxidative stress [59] and mediate heat–shock responses [60] probably through targeting the transcription factor DAF-16/FoXO [61], which can also be targeted by DHA according to our previous studies [19]. In fact, Zhou et al. recently showed that fluoxetine, a 5-HT reuptake inhibitor, was able to extend lifespan through modulating *sod-3* in a *daf-16*-dependent manner, since fluoxetine-induced lifespan extension was abolished in mutant nematodes with defective *sod-3* and *daf-16* [62]. Therefore, it seems reasonable to hypothesize that DHA-TG through protecting serotonergic neurons and maintaining their normal function, promoted oxidative stress resistance and increased the motility of 40Q nematodes, as summarized in Figure 8.

Recently, Menzel et al. reported that *C. elegans* requires ω-3 PUFA for aversive olfactory associative learning related to dopaminergic synapse and explained this phenomenon through the changes in the neuronal membrane fluidity [63]. Ω-3 LCPUFA phospholipids in the membrane disorganize the lipid rafts rich in cholesterol [64], thus increasing membrane fluidity, modulating signaling platforms and membrane receptors or transporters that conditionate cell migration and signaling transduction [65]. This effect is not only limited to nematodes but also is plausible in humans, as Zang et al. found that increased membrane fluidity prevents schizophrenia symptoms and is positively correlated with increased DHA contents [66]. To explain our results, we hypothesize that the effect of DHA in the serotonergic synapses might also be related with the impact of DHA in the membrane fluidity of neurons and sensitive cells. As nematodes do not use DHA as a substrate to directly synthesize specialized pro-resolving mediators [67] or endogenous metabolites, as mammals do, *C. elegans* rise a suitable model to study other functions of this molecule, such as its impact in cell membrane fluidity.

Current evidence suggests that patients suffering from HD, especially at the first stages, might benefit from ω-3 supplementation. Ω-3 LCPUFA has promising beneficial outcomes against the physical symptoms found in HD, such as sarcopenia [68], and also prevents the psychiatric symptoms very prevalent in HD patients, such as depression and irritability [15], caused by the reduced expression of 5-HT receptors in some regions of the brain [69,70]. In fact, it has been reported that EPA is able to recover serotonin levels in HD patients through replacing arachidonic acid (ω-6 LCPUFA) from neuronal membranes, which prevents the formation of the eicosanoid prostaglandin E_2_ (PGE_2_) and allows the release of serotonin [15]. Curiously, PGE_2_ is also present in nematodes [67]. Although the *C. elegans* nervous system is far from the complexity of the mammalian brain, we also found that an ω-3 oil rich in DHA-TG can improve the physical performance and serotonergic synapses of a polyQ nematode model. Overall, our findings might shed light on part of the mechanisms mediating the beneficial effects of ω-3 LCPUFA in HD patients.

## 4. Materials and Methods

### 4.1. C. elegans Strains and Maintenance

All the worm strains were maintained at 20 °C on standard NGM agar plates seeded with *Escherichia coli* OP50 as described elsewhere [71]. The WT *C. elegans* strain N2 (var. Bristol) and the mutant strains, AM141 (*rmIs133*[*unc-54*p::Q40::YFP]) and AM101 (*rmIs110*[F25B3.3p::Q40::YFP]), were obtained from the Caenorhabditis Genetics Center (CGC, University of Minnesota, Minneapolis, MN, USA), as was the *E. coli* feeding strain OP50. Both of the mutant strains were backcrossed to the WT background (Bristol N2) at least three times according to the references [37]. Synchronized *C. elegans* populations were obtained by dissolving the young adults in a sodium hypochlorite solution [72] before all the experiments. The obtained eggs hatched in M9 buffer (42.26 mM Na_2_HPO_4_, 22.04 mM KH_2_PO_4_, 85.56 mM NaCl, and 0.87 mM MgSO_4_) overnight and were transferred to new NGM plates, either control or treatment, the following day. The pre-fertile young adult worms were transferred to new plates containing 100 µM 5-fluoro-2-deoxyuridine (FUdR) when needed [73].

### 4.2. Preparation of DHA-TG Nanoemulsions

The nematodes were treated with an oil-in-water phase emulsion of DHA-TG as detailed in previous studies [19]. Briefly, DHA-TG oil (Brudy Technology, Barcelona, Spain) was mixed in M9 buffer with 20% of Tween 80 (Sigma-Aldrich Co., St. Louis, MO, USA) as the emulsifier [28]. The mix was vortexed for 5 min and sonicated (Q700, Qsonica Sonicators, Newtown, CT, USA) for 3 min, in 30 s intervals, to create nanoemulsions (amplitude = 60) according to Colmenares et al. [74]. The atmosphere of the tubes was replaced with nitrogen gas to avoid fatty acid oxidation. The DHA-TG oil was a novel formulation with targeted fatty acid positions in the TG.

The nanoemulsion stock solutions were diluted to the desired concentration using an M9 buffer with Tween 80 to standardize all the treatments at a 0.01% Tween 80 concentration. A vehicle treatment solution with Tween 80 at a final concentration of 0.01% in M9 was also prepared as detailed above. To feed the worms, the nanoemulsions were dispersed over the surface of agar plates containing a lawn of *E. coli* OP50, as described by Colmenares et al. [74]. The DHA solutions were freshly prepared and dried over the agar plates 1 h before the nematodes were transferred to the plates to avoid DHA oxidation. The amount of liquid added to the plates was constant and the same for all the concentrations. The nematodes were moved to fresh plates every 2–3 days depending on their feeding rate.

### 4.3. Lifespan Analysis

The lifespans of the nematodes were assessed as previously described by Lionaki and Tavernakis [75]. Briefly, the synchronized animals were transferred to NGM plates containing the vehicle or the treatments. The adults were scored manually and blindfolded as dead or alive every 1–2 days. More than 60 animals were analyzed per condition. The nematodes that ceased pharyngeal pumping and that had no response to gentle stimulation were recorded as dead. The worms that were males, that crawled off the plate, or that experienced non-natural death were excluded from the experiment.

### 4.4. Motility Assay

As in previous studies, motility was evaluated in the worms using the so-called thrashing assay which measured the swimming capacity of the nematodes in M9 buffer [19]. A thrash is defined as a change in the direction of bending at the middle of the body [52]. This assay is considered a measure of physical fitness that correlates with the healthspan in *C. elegans* [16]. Succinctly, the number of head thrashes was counted for 30 s per animal, and the average data were extrapolated to represent thrashes per min. Before scoring, each animal was acclimated in buffer for 30 s. At least 30 animals per condition were analyzed blindfolded. Each experiment was performed three times to obtain reproducible values.

### 4.5. Basal and Enhanced Slowing Response

Both basal slowing response and enhanced slowing response (BSR and ESR, respectively) are behavioral tests that give information about the synaptic connection of the nematode through feeding stimuli. Well-fed *C. elegans* have a fast rate of locomotion in the absence of food, and their motility slows down when food is present. BSR is mediated by dopaminergic neurons, specifically, cephalic (CEP), anterior deirid (ADE)m and posterior deirid (PDE) neurons, for which the sensory endings in the worm’s cuticle detect the presence of bacteria. Conversely, the ESR measures the associative learning related to the feeding condition, which is modulated by serotonergic neurons. For ESR measurement, the worms are shortly deprived of food, which makes them more sensitive to bacteria presence than in basal conditions. Therefore, they move more slowly in bacteria presence than in BSR. Mutant strains lacking serotonin have a defective ESR that can be rescued by the addition of exogenous serotonin; thus, ESR is supposed to be mediated by serotonergic neurotransmission, although the involved neurons are not fully identified [46].

BSR and ESR were performed as described by Sawin et al. [76]. Briefly, NGM plates with and without *E. coli* OP50 were used to count the body bends per 20 s. The locomotion rate was counted after 5 min of transfer to avoid overstimulation. More than 12 well-fed synchronized 4-day old adult worms per condition were tested. The assay was performed blindfolded, in two or more independent experiments. For ESR, the worms were starved for 30 min before testing.

### 4.6. Oxidative Stress Resistance Assay

The measures of oxidative stress resistance were performed according to Possik and Pause [77]. Summarizing, the wells were filled with 100 µL of M9 buffer and six 4-day old worms were transferred to each well. Then, 100 µL of 100 mM Paraquat (PQ) solution (methyl viologen dichloride hydrate, Sigma-Aldrich, St. Louis, MO, USA) were added to each well to a final concentration of 50 mM of PQ. Using the dissecting microscope, alive worms were scored every hour for 6 h. Looking at the worm shape, the tails, and the head movement at high magnification, the dead worms were differentiated from the alive worms. The plate was gently shaken before the count started. More than 30 worms were analyzed per condition in two independent experiments.

### 4.7. In Vivo Scoring of polyQ in Muscle Cells

The expression of the 40Q::YFP transgene produces aggregates of polyQ in an age-dependent manner, which can be followed using a dissecting microscope equipped with fluorescence. An extra treatment with Zn^2+^ at 50 µM in the NGM during the first 48h of life was included as a positive control [33,34]. The worms were mounted onto 2% agar pads and anesthetized with a drop of 0.5 M sodium azide. At least twenty adults (4 pictures with 5 worms) were analyzed for each condition; pictures were collected using a DM2500 (Leica, Wetzlar, Germany) vertical fluorescence microscope for scoring. The software Image J (1.54k version) was used for scoring the fluorescent 40Q aggregates [78].

### 4.8. RNA Extraction, Retrotranscription, and (RT)-qPCR

At 4 days of adulthood, 1000 worms per condition were harvested and resuspended in 300 µL of TRIzol™ (Invitrogen, Waltham, MA, USA) [79]. Then, the worms were frozen-thawed in liquid nitrogen 5–6 times and vortexed between cycles for preparing the lysates. Afterwards, the lysates were sonicated (Q700, Qsonica Sonicators, Newtown, CT, USA) in one cycle of 30 s (amplitude = 60) and centrifuged at 12,000 rpm at 4 °C for 15 min to collect the supernatant. The total RNA from the worms was isolated using Direct-zol™ RNA Miniprep (Zymo Research, Ivine, CA, USA) according to the manufacturer’s instructions. Before freezing at −80 °C, the RNA concentration was determined with a nanophotometer (Implen, Schatzbogen, Germany). Lastly, the cDNA was synthesized using a High-Capacity cDNA Reverse Transcription Kit (Applied Biosystems, Waltham, MA, USA).

For quantitative PCR analysis, 10 ng of the cDNA were mixed, with the oligonucleotide sequences used as primers and the LightCycler 480 SYBR Green I Master Mix (Roche, Basel, Switzerland), following the manufacturer’s instructions. The reaction was performed on the LightCycler^®^ 480 II (Roche, Basel, Switzerland) and the sequences used for the primers are listed in Table S1 (Supplementary Material). Each qPCR reaction was performed using more than three biological replicates in triplicate, and the qPCR levels were normalized to the expression of *act1*, which was predicted to be a structural constituent of the cytoskeleton. The fold change was normalized to that observed in vehicle *C. elegans* samples.

### 4.9. Statistical Analysis

The data were plotted as the mean ± SEM (standard error of the mean) of at least two individual experiments and three or more biological replicates. A statistical analysis was performed using GraphPad Prism version 8 for Windows (San Diego, CA, USA). The data were subjected to the Kolmogorov–Smirnov test for normality. For data with a normal distribution, a one-way ANOVA followed by Tukey’s or Dunnett’s test was used to compare treatments, whereas a two-way ANOVA followed by Dunnett’s post-test was used to compare three or more groups with two different independent variables. All the survival curves were analyzed with the Log-rank test. The statistical significance was determined as *p* < 0.05.

## 5. Conclusions

Summarizing, this study showed that treatment with a DHA-TG rich oil successfully prevented the nematodes from developing polyQ aggregate-associated dysfunctions, emphasizing the protection against serotonergic synaptic dysfunction. Since polyQ aggregates in humans are associated with the etiology of HD and SCA, among others, our study reinforces the potential of DHA for the prevention of polyQ-related disease development, in addition to contributing, with our data, to the elucidation of the mechanistic effect of DHA in the oxidative stress resistance capacity, the synaptic function, and the protein aggregates reduction in polyQ models.

## Data Availability

The data presented in this study are available in this article (and in Supplementary Materials). Additionally, other items that support the results of this study will be made available upon reasonable request.

## Supplementary Materials

The supporting information can be downloaded at https://www.mdpi.com/article/10.3390/ijms252312594/s1.
