# Supplementary material for: Docosahexaenoic Acid (DHA) Supplementation in a Triglyceride Form Prevents from Polyglutamine-Induced Dysfunctions in Caenorhabditis elegans
Source: Int J Mol Sci. 2024 Nov 23;25(23):12594. doi: 10.3390/ijms252312594 (PMC11640917; doi:10.3390/ijms252312594)
Supplement: Supplementary file 1 [file ijms-25-12594-s001.zip › ijms-3326524-supplementary.pdf]

Supplementary material

Docosahexaenoic acid (DHA) supplementation in a triglyceride form alleviates polyglutamine-aggregates associated dysfunctions in *Caenorhabditis elegans*

Ignasi Mora [1,2,*], Alex Teixidó [3], Rafael P. Vázquez-Manrique [4,5,6], Francesc Puiggròs [7,*] and Lluís Arola [8]

[1] Brudy Technology S.L., 08006 Barcelona, Spain
[2] Universitat Rovira i Virgili, 43003 Tarragona, Spain
[3] Eurecat, Centre Tecnològic de Catalunya, Nutrition and Health Unit, 43204 Reus, Spain
[4] Laboratory of Molecular, Cellular and Genomic Biomedicine, Instituto de Investigación Sanitaria La Fe, 46026 Valencia, Spain
[5] Joint Unit for Rare Diseases IIS La Fe-CIPF, 46012 Valencia, Spain
[6] Centro de Investigación Biomédica en Red de Enfermedades Raras (CIBERER), 28029 Madrid, Spain
[7] Eurecat, Centre Tecnològic de Catalunya, Biotechnology Area, 43204 Tarragona, Spain
[8] Nutrigenomics Research Group, Departament de Bioquímica i Biotecnologia, Universitat Rovira i Virgili, 43007 Tarragona, Spain
* Correspondence: Dr. Francesc Puiggròs (francesc.puiggros@eurecat.org)

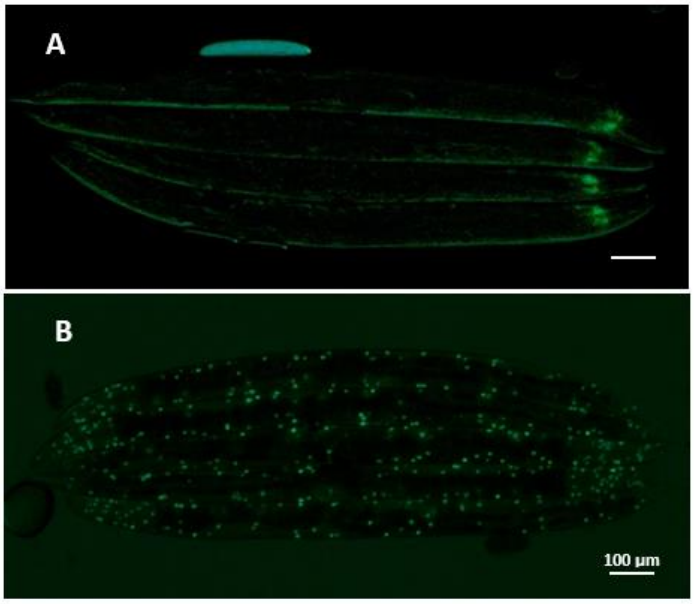

Figure S1. Images of 40Q *C. elegans* strains used in the study. (A) Photo of 4 nematodes with fluorescent pan-neuronal 40Q aggregates, AM101 (rmIs110[F25B3.3p::Q40::YFP]). (B) Photo of 5 nematodes with fluorescent muscular 40Q aggregates, AM141 (rmIs133[unc-54p::Q40::YFP]). Worms were mounted onto 2% agar pads and anesthetized with a drop of 0.5 M sodium azide. Images were taken using a DM2500 (Leica, Wetzlar, Germany) vertical fluorescence microscope.

Supplementary material

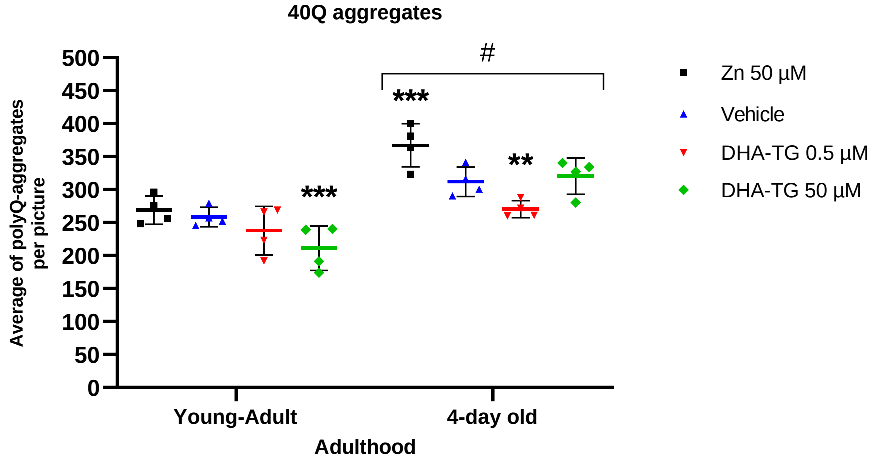

Figure S2. Number of 40Q aggregates of nematode strain AM141 *rmIs133*[unc-54p::Q40::YFP]) after treatments. Data shows a line expressing the average of polyQs ±SD counted in 4 different pictures (4 points) per treatment. Each picture had 5 worms, total n=20. Fluorescent 40Q aggregates were counted with ImageJ. Differences were considered significant at $p < 0.01$ (**) and $p < 0.001$ (***) after two-way ANOVA followed by Sidak's test.

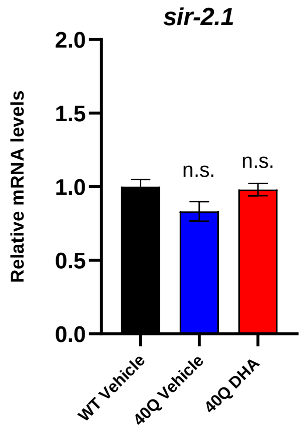

Figure S3. Gene expression levels of *sir-2.1* in nematode strain AM101 at 4 days of adulthood after DHA-TG 0.5 µM treatment expressed vs WT. Differences compared to 40Q Vehicle were considered significant at $p<0.05$ after one-way ANOVA followed by Tukey's test (n.s.= not significant).

Supplementary material

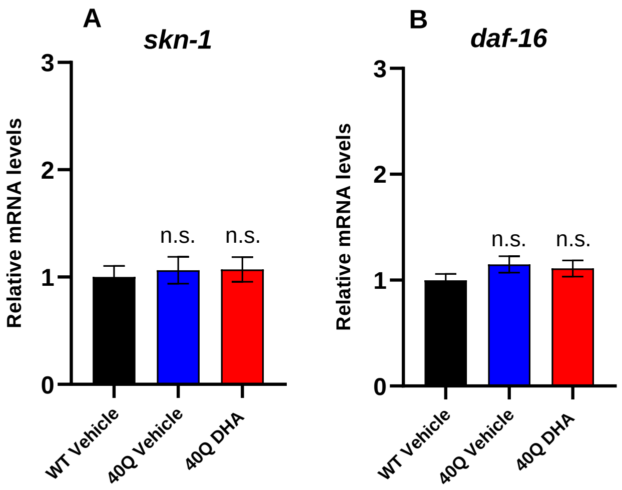

Figure S4. Gene expression levels of *skn-1* (A) and *daf-16* (B) in nematode strain AM101 at 4 days of adulthood after DHA-TG 0.5 µM treatment vs WT. Differences compared to 40Q Vehicle were considered significant at p<0.05 after one-way ANOVA followed by Tukey's test (n.s.= not significant).

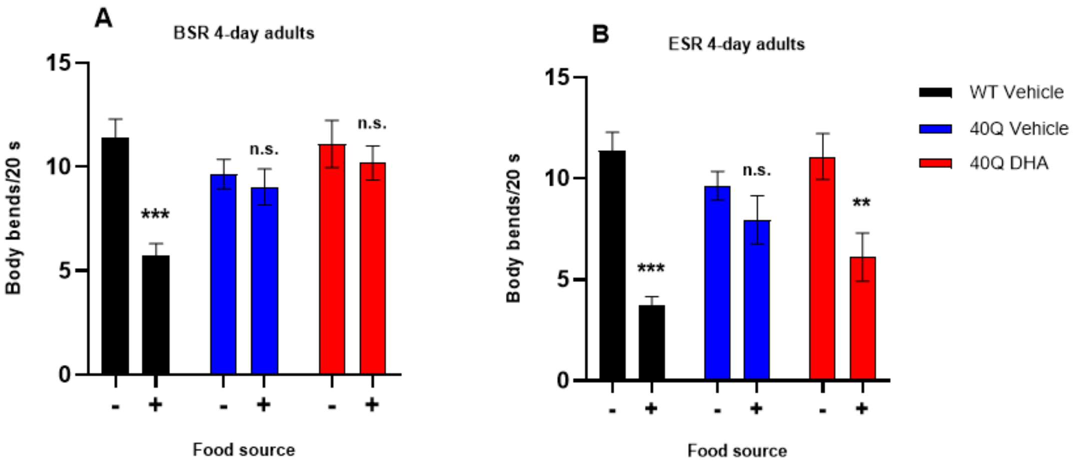

Figure S5. BSR (A) and ESR (B) of nematode strain AM101 at 4 days of adulthood after DHA-TG 0.5µM treatment represented as body bends per 20 s. Statistical differences compared to Food source (-) group were considered significant at p<0.05(*), p<0.01(**) and p<0.001(***) after one-way ANOVA followed by Tukey's test (n.s.= not significant).

Supplementary material

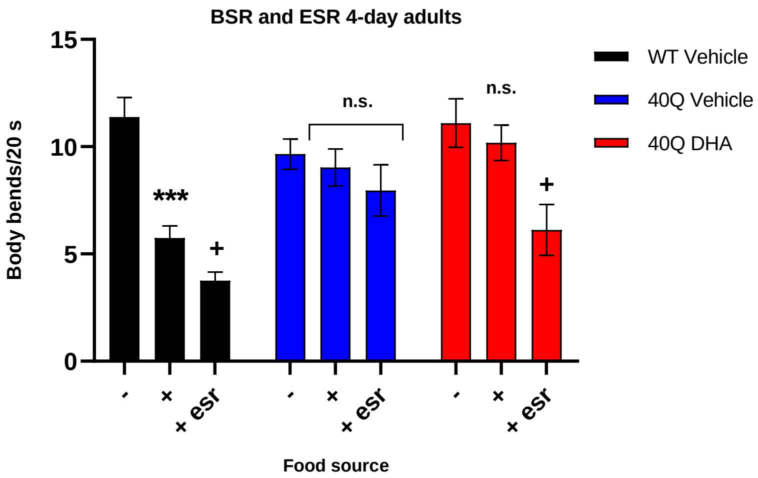

Figure S6. Differences between BSR and ESR of nematode strain AM101 at 4 days of adulthood represented as body bends per 20 s. Statistical differences compared to Food source (-) group were considered significant at *p*<0.001(***) and differences compared to Food source (+) group were considered significant at *p*<0.05(+) after two-way ANOVA followed by Sidak's test (n.s.= not significant).

Table S1. Oligonucleotide sequences used as primers in qPCR

Gene (Wormbase accession nº)	Encoding	Primers sequences (5' → 3')
sod-3 (C08A9.1)	Mitochondrial superoxide dismutase (Mn-SOD)	F- GGCTAAGGATGGTGGAGAAC<br>R- ACAGGTGGCGATCTTCAAG
skn-1 (T19E7.2)	Nuclear factor erythroid 2-related factor 2 (NRF2)	F- GTTCCCAACATCCAACTACG<br>R- TGGAGTCTGACCAGTGGATT
sir 2.1 (R11A8.4)	Sirtuin 1 (SIRT 1)	F- TGGCTGACGATTCGATGGAT<br>R- ATGAGCAGAAATCGCGACAC
aak-2 (T01C8.1)	AMP-Activated Kinase (AMPK)	F- TGCTTCACCATATGCTCTGC<br>R- GTGGATCATCTCCCAGCAAT
daf-16 (R13H8.1)	Forkhead box O (FOXO)	F- TCAGGGATAAGGGAGATTCG<br>R- CAGATTGTGACGGATCGAGTT
dat-1 (T23G5.5)	Dopamine transporter (DAT)	F- CGTACTCGGCTACATGTCCTGCAA<br>R- CTGAAAGGCCGGTGATGATAGCTTC
cat-1 (W01C8.6)	Vesicular monoamine transporter (VMAT)	F- CTTGCTCTCTTCGATGGATCAA<br>R- ATTTCCGATGGTGATTGCTCCT
mod-5 (Y54E10BR.7)	Serotonin transporter (SERT)	F- AAAGCACCGACAGCATCAG<br>R- TAAGAAAAGCACCGCCACC
ser-4 (Y22D7AR.13)	Serotonin receptor 1A (5-HT_{1A})	F- CGTCTGCACTGCCTCAATTC<br>R-AAACCCCTCATCCTTCCACC

Supplementary material

tph-1 (ZK1290.2)	Tryptophan hydroxylase (TPH)	F- AAGAGGCCCAGCAGAAACTC<br>R- CAGGTTGATGTCTGAGCGGA
act-1 (T04C12.6)	Actin	F- TCGGTATGGGACAGAAGGAC<br>R- CATCCCAGTTGGTGACGATA
